# The Human Odor

**Published:** 1900-02

**Authors:** 


### THE HUMAN ODOR.

THE fact that some animals, notably the dog, can recognize the presence of their master or a friend by the odor leads to the belief that every human being has a distinctive odor more or less constant. The sense of smell in man is not sufficiently developed to enable him to differentiate by this means between members of his own species; but in animals in which the high development of the sense organs is essential to their existence in a natural state, this method of recognition is easy.

It is entirely reasonable to presume that as no two individuals look alike, none smell alike. The smell of the negro is highly and frankly characteristic. It is more marked in the pure negro than in the mixed race, where the heavy ammoniacal odor gives place to a faint nauseating scent. Surgeon Parke, who accompanied the Emin Pasha Relief Expedition, states that the pigmies are the most odoriferous of all negro tribes. He describes the effluvium that emanates from the body of a female pigmy when excited as overpowering. This observation is often made in the South, that small black negro women are especially gifted in respect of their smell, which approaches in intensity and similarity that of the scavenger beetle, which owes its pungency to ammoniacal decomposition of its food, in which it buries itself to fashion its globe.

It is possible that the sense of smell in man may be highly educated or congenitally exceeding acute, as in the following case cited by Dr. Bett (*Archiv der Gesammter Physiologie*), quoted in the *National Druggist:* The doctor states that a friend of his, with bandaged eyes and every precaution against collusion, was enabled, by the sense of smell alone, to recognize persons with whom he was acquainted, and to call their names the moment that they came into the room and at the distance of several paces. The experiments were varied in a number of ways, but with the unerring faculty of the bloodhound this man detected the identity of every individual presented. Other instances of a similar keenness of scent are cited by Dr. Bett. According to the man who gave the exhibition, every family has a characteristic odor, common to all the members thereof, but the intensity of which usually varies sufficiently among the various members to enable him to distinguish each individual member.

